# Delivering of Proteins to the Plant Vacuole—An Update

**DOI:** 10.3390/ijms15057611

**Published:** 2014-05-05

**Authors:** Cláudia Pereira, Susana Pereira, José Pissarra

**Affiliations:** 1 Institute for Molecular and Cell Biology (IBMC), University of Porto, Rua do Campo Alegre, 823, 4150-180 Porto, Portugal; E-Mail: claudia.pereira@ibmc.up.pt; 2 Department of Biology, Faculty of Sciences, University of Porto and Centre for Biodiversity, Functional and Integrative Genomics, Rua do Campo Alegre, s/n, FC4, 4169-007 Porto, Portugal; E-Mail: jpissarr@fc.up.pt

**Keywords:** plant vacuolar protein sorting, vacuolar sorting determinants, specialization of the trafficking pathways, plant-specific insert, Golgi-independent route

## Abstract

Trafficking of soluble cargo to the vacuole is far from being a closed issue as it can occur by different routes and involve different intermediates. The textbook view of proteins being sorted at the post-Golgi level to the lytic vacuole via the pre-vacuole or to the protein storage vacuole mediated by dense vesicles is now challenged as novel routes are being disclosed and vacuoles with intermediate characteristics described. The identification of Vacuolar Sorting Determinants is a key signature to understand protein trafficking to the vacuole. Despite the long established vacuolar signals, some others have been described in the last few years, with different properties that can be specific for some cells or some types of vacuoles. There are also reports of proteins having two different vacuolar signals and their significance is questionable: a way to increase the efficiency of the sorting or different sorting depending on the protein roles in a specific context? Along came the idea of differential vacuolar sorting, suggesting a possible specialization of the trafficking pathways according to the type of cell and specific needs. In this review, we show the recent advances in the field and focus on different aspects of protein trafficking to the vacuoles.

## 1. Introduction

Plants play an important role in the world economy as they represent the major source of food, but also of textiles, building materials, bio-fuels and medicinal products. In the past decades, plants have also been exploited for the production of recombinant pharmaceutical products, such as edible vaccines, antibiotics and vitamins. Plant biologists are currently joining efforts for plant science to progress toward of a definition of integrated circuits of metabolic pathways and sub-cellular structures to accompanying the challenges imposed by animal and yeast sciences.

Besides understanding plants as a whole organism, it is fundamental to track the subcellular mechanisms beneath. Identifying the molecules (proteins and enzymes), their biogenesis and their functions is fundamental to understand their role in plant cell homeostasis and ultimately to develop rational crop improvement strategies. The plant cell is effectively an integrated community of membrane-delimited compartments, each of which has evolved to optimize and separate specific biochemical functions. Functional organization of the endomembranes is a key cellular infrastructure allowing the cell the continuous adaptation needed for the metabolic and structural changes occurring in vegetative and reproductive tissues. The major production line of the cell biosynthetic factory is made by distinct organelles constitutive of the cell secretory pathway: Endoplasmic Reticulum, Golgi Apparatus, Endosomes, Pre-vacuolar and Vacuolar compartments. The specificities of plant endomembrane biology, notably the Endoplasmic Reticulum-Golgi complex or the endosomal and vacuolar organization, outline the amazing ability of plant cells to re-organize its membrane system according to the cell needs.

Presently, studies on plant endomembrane focus on several lines of research: the functional characterization of compartments designing the endomembrane system, the identification of pathways and vesicle carriers to transport cargo molecules from one compartment to the other and the identification of molecular machineries associated with these transport specificities. Any protein trafficking within the endomembrane system is orchestrated by all the events, permitting to reach the right compartment, by the right route, at the right time.

## 2. Plant Vacuoles

In most of the plant cells, the vacuole is the largest structure present and plays several roles in maintaining organization and cellular function and is involved in intracellular digestion of various materials, maintaining of turgor and accumulation of nutrients, ions and secondary metabolites. In seeds, protein storage vacuoles (PSVs) are the most abundant whereas in vegetative tissues the vacuolar functions are accomplished by large central vacuoles denominated lytic vacuoles (LV) [1,2]. The plant vacuolar system can become quite complex as in the same cell can co-exist two types of functionality distinct vacuoles [3]. Initially it was accepted that each type of vacuole was characterized by the type of aquaporin present in the tonoplast—tonoplast intrinsic proteins (TIPs): γ-TIP, lytic vacuole and α-TIP, protein storage vacuole [4,5]. However, the real situation can be much more complicated since overlap of both markers has been observed in vacuolar remodeling during transition between distinct types of vacuoles [6,7].

Vacuolar development and remodeling, particularly during seed germination, has gained a notorious importance given the immense changes occurring in a short time. It has been proposed that vacuolar membranes of PSV are transformed into those of central vacuoles upon germination [8]. Findings in soybean and pumpkin have shown that during germination the PSV undergoes an important dedifferentiation with gradual degradation of α-TIP, internalization of the tonoplast and formation of a new tonoplast carrying vacuolar H^+^-ATPase, V-PPase and γ-TIP [8,9]. However, this rearrangement of the vacuole seems to be related to the cell type, and eventually its specialization, since Olbrich and collaborators [6] observed that in meristematic cells a single vacuole type is formed initially presenting characteristics of both PSV and LV, that is gradually transformed into a central vacuole as the differentiation proceed. Recently, Bolte and colaborators [7] described the remodeling of *Arabidopsis thaliana* vacuome upon germination, illustrating the complexity of this system. Their results demonstrate a rapid and extreme reorganization of PSVs and LVs during early stages of seed germination, with the coexistence of two distinct compartments with very distinguishable roles.

The vacuolar metabolome has gained interest recently as the preliminary results obtained provide the basis for future studies to compare the storage and lytic metabolomes that would allow a more comprehensive understanding of the vacuolar systems of transport and metabolism [10].

## 3. Routes to the Vacuole

Vacuolar sorting is nowadays and since the last decade one of the most advanced and attractive research field in what concerns secretory protein trafficking. Proteins inserted in the Endoplasmic Reticulum (ER) have different ways to reach the vacuole, and may use different halt-stations to do so. For instance they may go through the Golgi Apparatus (GA), or bypass the GA, they may reach the plasma membrane and come back to the vacuole or they may be stored in intermediate compartments before to be delivered. From the trans-Golgi network (TGN), transit routes to the cytoplasmic membrane and transport to the vacuole diverge. Proteins lacking a signal enter the so-called default pathway, being transported in vesicles that eventually fuse with the plasma membrane, releasing their contents to the outside [11]. Vesicle trafficking on the far end of the GA is more complex and with more intervenients than on its *cis*-face as there is a protein sorting triangle between TGN, vacuole and endosome/plasma membrane. The number of SNARE proteins existing in the trans-GA illustrates the high differentiation degree and the large number of specific membrane-recognition events possible [12]. Secretory and endocytic trafficking pathways intersect in post-Golgi compartments of the endomembrane system. The post-Golgi compartments are considered morphologically distinct: trans-Golgi network/early endosome, multivesicular body/prevacuolar compartment/late endosome and two types of vacuole [13,14]. Multivesicular bodies (MVBs) act as a prevacuolar compartment (PVC) in route to the LVs, thus functionally corresponding to the late endosome (LE) of animal cells. It is generally accepted that MVBs fuse with the tonoplast, releasing their membrane-bound cargo into the vacuole [14]. Recently, it was proposed the existence of an intermediate compartment between the PVC and the LV in tobacco leaf epidermis [15], but more studies are needed to confirm its localization and function.

Conversely to the other routes described, vacuolar sorting of secretory proteins requires positive sorting at the TGN. The cargo proteins targeted to the vacuole are recognized at the GA level by vacuolar sorting receptors (VSRs) and are released into PVC/MVB after fusion of the transport carriers with their membrane. The VSR is then recycled back to the TGN and the cargo molecules are transported to the vacuole, where the fusion of the PVC/MVB with the tonoplast occurs [15]. As the trafficking to the vacuole can occur by different routes and may involve different intermediates it will be approached in more detail in the next section.

In a simplified version of protein transport to the vacuolar system two post-Golgi routes can be considered, ending in different vacuolar compartments—lytic or protein storage—being closely related to the type of signal present in the protein (*N*- or *C*-terminal signal) [5,16]. However, the existence of direct routes from the ER to the vacuoles has been demonstrated and may represent a general mechanism of vacuolar ontogeny in plant seeds [17]. In the GA-mediated pathway the proteins leave the ER in coat protein complex II (COPII) vesicles to the GA and from here are transported to the vacuole via the prevacuolar compartment (PVC). Studying storage proteins, additional pathways bypassing the GA were discovered in seeds: the proteins can be directly delivered to the vacuole in large vesicles that bud from the ER or leave the ER in KDEL vesicles (KV) that eventually fuse to the PSV [4,17]. Contrarily to the initial observations, direct ER-to-Vacuole transport is not limited to storage proteins being already described for tonoplast aquaporins and processing enzymes [18,19].

Transport to the lytic vacuole occurs after recognition of the sorting signal by the receptor BP-80 (binding protein of 80 kD), a membrane protein responsible for packaging the cargo-protein in clathrin-coated vesicles for transport to the pre-vacuolar compartment and, finally, to the lytic vacuole. Other proteins, with a *C*-terminal signal or a signal in the middle of the protein sequence, are transported to the vacuole in dense vesicles derived from the Golgi complex. In a less studied route, observed in storage organs, proteins do not pass through the Golgi apparatus and are transported to the vacuole in vesicles derived from the endoplasmic reticulum (precursor-accumulating vesicles) [16,19] Recently, a study involving human α-mannosidase showed that this protein is able to reach the plant vacuole in the absence of the mammalian mannose-6-phosphate receptors, required for its transport in animal cells, and trough a GA-independent route suggesting the existence of direct ER-Vacuole pathway [20]. At the same time, another study focusing the aspartic proteinase cardosin A also showed a GA-independent route in tobacco leaves, mediated by an internal signal [21]. Despite this new evidence of a GA-independent route to the lytic vacuole, the mechanisms behind the sorting at the ER level and the intracellular compartments involved are still unclear.

Recently, Stigliano *et al.* [22] used two vacuolar fluorescent reporters, glycosylated-GFP fusions with the *C*-terminal Vacuolar Sorting Determinant (VSD) of tobacco chitinase and an *N*-terminal fusion with the sequence-specific VSD of the barley aleurain demonstrating a differential Endoglycosidase H (Endo-H) sensitivity and therefore differential glycan modifications of the glycosylated GFPs. Their finding suggests two different and independent routes to the vacuole for the two reporters and confirmed that GFPChi transport from the ER to the vacuole is not fully dependent on the Golgi apparatus even if the identity of the compartments through which GFPChi transit on their way to the vacuole could not be established. The characterization of intermediate compartments may be further complicated by evidences that new types of compartments may be specifically induced in some circumstances as described for *Arabidopsis* plants exposed to carbon starvation [23].

The issue of multiple vacuolar transport routes has been already addressed for tonoplast proteins. Information on tonoplast proteins being delivered via three different transport routes was compiled by Robinson and Pimpl [24]. Recent evidence showed that a direct transport route to the tonoplast from the ER for some membrane proteins occurs during vacuole biogenesis in post-meristematic cells. Vacuolar proton ATPase subunit a3 (VHA-a3) exits the ER in a COPII-independent manner and successfully reaches the tonoplast when ER to Golgi transport is blocked [25]. The authors describe a model of vacuole biogenesis in that the ER could be the main membrane source for the biogenesis of the plant LV and point that evidence is accumulating for direct ER-to-vacuole transport and a possible, although transient, attachment of these structures [26].

## 4. Sorting Signals

For all eukaryotic cells, the transport from the Golgi apparatus to the vacuole or lysosomes requires positive information from the protein. In mammals, the signal to transport proteins to the lysosome is the addition of a mannose 6-phosphate residue during the passage through the Golgi complex, while in plant cells, the vacuolar sorting is part of the peptide sequence [5,27]. Several vacuolar sorting signals/vacuolar sorting determinants (VSS/VSD) have been described in plants and they can be divided into three major groups. The sequence specific sorting signals (ssVSD) require a conserved sequence of Asn-Pro-Ile-Arg (NPIR) or similar, which do not tolerate significant changes. Usually this type of signal is located at the *N*-terminal of the protein directed to the lytic vacuole. The second main group comprises the signals present at the *C*-terminal region of the protein (ctVSD) and normally designates proteins destined to the protein storage vacuole. These signals do not have any homologous sequence or size set, having in common that they are rich in hydrophobic amino acids and need to be exposed at the *C*-terminus to operate. Finally, the third group corresponds to signals dependent on the tertiary (physical) structure of proteins (psVSD) and is most common in storage proteins. These sorting signals are present in the protein sequence, and may correspond to more than one polypeptide that function as whole when the protein acquires the native conformation [11,27,28].

For a peptide to be considered a true vacuolar sorting signal it must be both necessary and sufficient for this function [5,29]. There are some described examples of vacuolar sorting determinants that satisfy these two assumptions [4,27,30,31]. However, protein sorting to the vacuole and the issue of the efficiency of a single VSD is questioned by data showing the existence of two types of VSD in the same protein. It was shown that the *C*-terminal region of alpha-subunit of soybean beta-conglycinin has two sorting signals, one of the ctVSD type and other of the sequence-specific type [32]. Both of them function for trafficking to the PSVs, but it is not clear if both use the same VSR. Another recent study showed the existence of two VSDs in the same protein with different properties: a typical ctVSD and an unconventional vacuolar sorting domain, the PSI (Plant Specific Insert), both part of cardosin A sequence [21]. The plant specific insert (PSI) is an extra protein domain of approximately 100 amino acids present in most plant aspartic proteinases (AP) precursors and is usually removed during the proteolytic maturation of these proteinases. The PSI is highly similar to saposins and saposin-like proteins (SAPLIPs) and the proposed role of the PSI in the targeting of plant APs to the vacuole resembles what has been described for the association of mammalian saposin C and cathepsin D. The formation of the prosaposin–procathepsin D complex could explain the proposed M6P-independent lysosomal targeting of cathepsin D mediated by saposin C (reviewed in [33]). Pereira *et al.* demonstrated that cardosin A is still able to reach the vacuole without one of the VSDs (the typical ctVSD and the unconventional vacuolar sorting domain PSI) and that each of them alone is able to redirect a normally secreted mCherry protein to the vacuole [21]. The existence of two VSDs in the same protein, although raising the question of their sorting efficiency, can be envisaged as a specific manner that plant cells developed to regulate vacuolar targeting in different tissues and physiological stages. On the other hand, as described before for two different storage proteins carrying two VSDs, the cell can benefit from a cumulative effect of the sorting signals [32,34].

Additionally, other study carried out by Pompa *et al.* [29] with the storage protein phaseolin showed that a tetrapeptide at the *C*-terminus is required for proper vacuolar sorting. The authors provided evidence that the *C*-terminal segment of a phaseolin polypeptide undergoes homotypic interactions that allow the formation of a disulfide bond when a Cys residue is introduced proximal to the AFVY *C*-terminal vacuolar sorting signal. Furthermore, the authors claimed that the vacuolar sorting signal of phaseolin is not necessary for those homotypic interactions and its sorting function can be partially replaced by a *C*-terminal Cys residue. Based on this data, the importance of multiple copies of vacuolar determinants in assembled homo-oligomeric storage proteins was discussed and is far from being clear. The ratio between number of vacuolar sorting determinants and the size of the passenger protein was also proposed to play a role in the sorting efficiency [32]. In light of the recent advances in protein sorting to the vacuole, it is clear that the initial picture proposing three types of VSD directing proteins to a specific type of vacuole is not completely accurate and more studies are needed to clarify this aspect

## 5. A Role for Glycosylation

In plants, the Golgi apparatus (GA) is a central key in relation to the synthesis of cell wall polysaccharides, and of glycolipids and glycoproteins for the cellular membranes. It has thus an important role in the modification and targeting of the protein and is involved in folding, processing and storage of proteins. It has been long discussed the role of the glycosylation in the vacuolar routes taken by proteins in the secretory pathway, in particular involving a GA bypass [35,36]. Studies from Wilkins and co-workers [37] suggested in the early 90s that protein glycosylation was not required per se for vacuolar trafficking, but could affect processing and transport rates of cargo along the vacuolar route [37]. It is largely accepted that typical storage proteins like 7S and 2S globulins accumulate in PSVs in a GA-independent manner, through precursor accumulating vesicles (PAC vesicles) [38]. Although several examples of GA bypass can be found in maturing seeds because of the specificity of being storage structures, in leaf tissues the examples are scarcer and despite being a pertinent issue among plant vacuolar trafficking community, no clear data on the role of GA glycosylation has been reported, but a few novel studies shed some light on the subject. Recently it was shown that the glycosylation status of a newly described vacuolar sorting determinant—aspartic proteinase cardosin A PSI—could interfere with the route taken by the protein to the vacuole, but would not impair its localization in leaf cells [21]. The authors showed that replacing the PSI domain of cardosin A (not glycosylated) with the PSI domain of cardosin B (glycosylated) changes the PSI mechanisms of vacuolar sorting, as it shifted from a COPII-independent trafficking pathway to a COPII-dependent trafficking pathway [21]. Given that cardosin A may follow two different vacuolar routes, it is expected that the glycosylation pattern of the protein bypassing the Golgi apparatus is considerably different from the one passing through Golgi–TGN–PVC which raises challenging questions since *N*-glycans confer important *in vivo* functions to glycoproteins, namely in cell signaling. Presently, little is known about the glycosylation pattern of plant vacuolar proteins. Stigliano and co-workers [22] developed a new set of glycosylated GFP vacuolar markers that provided evidences for the existence of a transport route to the vacuole independent of the GA. Therefore, vacuolar proteins bypassing the Golgi apparatus are expected to evidence marked changes on the glycosylation patterns.

The existence in plants of a new type of vacuolar traffic that can be used by leaf cells to transport vacuolar proteins offers a great potential, in particular for recombinant protein production in the near future. The available studies in this subject point to a stunning increase in protein production when it is targeted to the vacuole. The problem of targeting foreign proteins to the vacuole is that once entering the secretory pathway, proteins undergo post-translation modifications such as glycosylation. Despite plants produce the same glycosylation patterns as mammals in the ER, when entering the GA the trimming of the glycans is sufficiently different to cause immunogenicity risks. Plant systems with modified glycosylation pathways lacking potentially immunogenic *N*-glycans are useful “pharming” systems to produce biopharmaceuticals with reduced allergenicity. Therefore, the targeting of therapeutic proteins to the vacuole bypassing the GA would be an exciting approach to overcome the plant-specific glycosylation problem.

## 6. Specialization of Vacuolar Trafficking Pathways: The Particular Case of Cardosins

A new topic of discussion is arising in what concerns the vacuolar routes some proteins take in specific organs and how those routes changes in organs with a different metabolic activity. It was observed that the same protein can be either secreted to the apoplast or directed to the vacuole, or accumulate in PSVs or LVs traveling though different routes, depending on the cell type and developmental stage suggesting a tight mechanism of regulation of trafficking.

Cardosins, an Aspartic Proteinase family identified in cardoons, illustrate such plurality and complexity in sorting mechanisms [21,39]. In the floral tissues of *C. cardunculus*, cardosin A is mainly found in the protein storage vacuoles of the stigmatic papillae while cardosin B is found in the extracellular matrix of both stigma and style transmitting tissue [40–42]. In heterologous systems, Cardosin A was found to accumulate in different types of vacuoles according the tissue type: lytic vacuoles in epidermal leaves, and protein storage vacuoles in *Arabidopsis thaliana* seedlings. It has also been shown that preprocardosin A is processed along the vacuolar pathway and the last processing step to its mature form, including the removal of the internal PSI domain, occurs in the vacuole probably due to its acid environment [40]. However, in cardoon seeds an unprocessed precursor form of cardosin A, reaches the protein body (PB) in a GA-independent manner [19]. Processed cardosin A is subsequently detected in the central lytic vacuole as it engulfs PBs. The dual localization of cardosin A in protein bodies in cardoon cotyledon cells, and in protein storage vacuoles in flower cells [19], could be related to the existence of a PSI specific pathway, dependent on the type of tissue as recently proposed by Pereira *et al.* [21]. The authors postulate an alternative, COPII independent, vacuolar route mediated by non-glycosylated PSI domains such as the PSI of cardosin A and that this PSI-mediated pathway might be more relevant in metabolically active organs (such as flowers and seeds), where protein storage vacuoles are predominant over lytic vacuoles, and cells have different needs. Pereira and collaborators [21] demonstrated that a typical *C*-terminal VSD but also an unconventional PSI signal of cardosin A are sorting determinants and mediate two different trafficking pathways. In the experimental model used, the *C*-terminal signal is dominant over the PSI signal, as it dictates the route the protein takes to the vacuole. It is likely that the two signals act independently and provide the APs with a functional plasticity, controlling vacuolar traffic according to cell constraints or needs. Particularly relevant is the new insight into the regulatory function of the PSI domain in aspartic proteinase trafficking through a specific PSI signaling pathway, which may operate in parallel with another vacuolar pathway.

It was described a Golgi-independent route in which proteins can be transported directly from the ER to the LV by means of entities such as plant-specific endoplasmic reticulum bodies and authors argue that it could provide plants a means for rapid adaptation to environmental changes [43]. Indeed, the existence of plant-specific alternative pathways for proteins to reach the lytic vacuole and the protein storage vacuole appear as adaptive mechanisms providing the flexibility required for plants to cope with constantly changing developmental and environmental stimuli and a variety of stress challenges.

Duarte and collaborators [40] demonstrated that cardosin A, is correctly targeted to the vacuoles when expressed in heterologous systems such as tobacco leaf epidermis and *Arabidopsis* seedlings. However, the authors also observed the presence of endoglycosidase-H-sensitive intermediate forms of cardosin A and the failure of the dominant-inhibitory Rab GTPases to completely inhibit processing of procardosin A. This was an early evidence of the existence of two vacuolar routes for cardosin A, one of them bypassing the GA as was subsequently demonstrated by Pereira and collaborators [21]. Moreover Duarte *et al.* observed that an intermediate form of cardosin A is secreted from tobacco protoplasts and raised the hypothesis that cardosin A is first secreted as an intermediate processing form and targeted to the vacuole only after subsequent endocytosis from the apoplast. If there is indeed a PSI-mediated route of procardosina A to the cell wall and subsequent endocytosis to the vacuole remains to be proved.

De Caroli *et al.* studying the secretion patterns of cell-wall proteins in tobacco protoplasts and leaf epidermal cells, had observed that a protein fusion of polygalacturonase inhibitor protein, PGIP2–GFP reached the cell wall by passing through ER and Golgi stacks but was not stably retained in the cell wall due to internalization to an endosomal compartment and later to the vacuole. However, stable localization of PGIP2 in the cell wall was observed in the presence of a specific fungal endopolygalacturonase ligand in the cell wall. The authors describe cell wall-directed secretion routes distinguishable from the bulk-flow default secretion pathway labeled by secGFP and suggest that rigorous and more complex pathway controls than the simple mechanism of bulk flow are the basis of cell-wall growth and differentiation [44].

The observed presence of the unprocessed precursor form of cardosin A in the cell walls of specific cell types [19,41,45] also points to a more specific regulated route of this form to the plasma membrane/cell wall complex where it may interact with other proteins that may sense specific events related to development or stress or otherwise be internalized to the vacuole via endocytosis.

Events that occur at the cell wall level in response to cell transition states (such as seed germination or pollen pistil interactions) or pathogen attack may involve sensing and signaling molecules that could somehow trigger cardosin A (and most probably many other proteins with similar characteristics), an otherwise vacuolar protein, to the apoplast.

Proteins or peptides with predicted intracellular function or experimentally established intracellular localization are also secreted to the apoplast under certain conditions, or in specific tissues [46]. Some mammalian proteins that usually have an intracellular function are secreted following specific signals and in specific tissues and secretion can be rapid and activated by external stresses (reviewd in [46]). Possible explanations for alternative secretory routes are advanced including the possibility that posttranslational modifications may not be desirable for some proteins in specific contexts [46].

Mechanisms of unconventional secretion have been identified for proteins that initially enter the secretory pathway through ER translocation and are secreted in a Golgi-independent manner, and also for “leaderless” cytoplasmic proteins lacking the presence of a signal peptide [47,48]. Possible pathways of unconventional protein secretion (UPS) in plants have been recently reviewed ([47,48]) and include both MVB and vacuole fusion with PM in response to pathogen attach, Golgi independent secretion of cytoplasmic proteins and secretion through recently discovered exocyst-positive double membrane organelles (EXPO).

The contribution of the different pathways postulated for cardosin A depends on cell type and cell requirements. Some pathways may be found in most, if not all, cell types, while others are associated with the specialized functions of particular cell types, and cell identity transitions and general changing developmental and environmental conditions.

**Figure 1.**
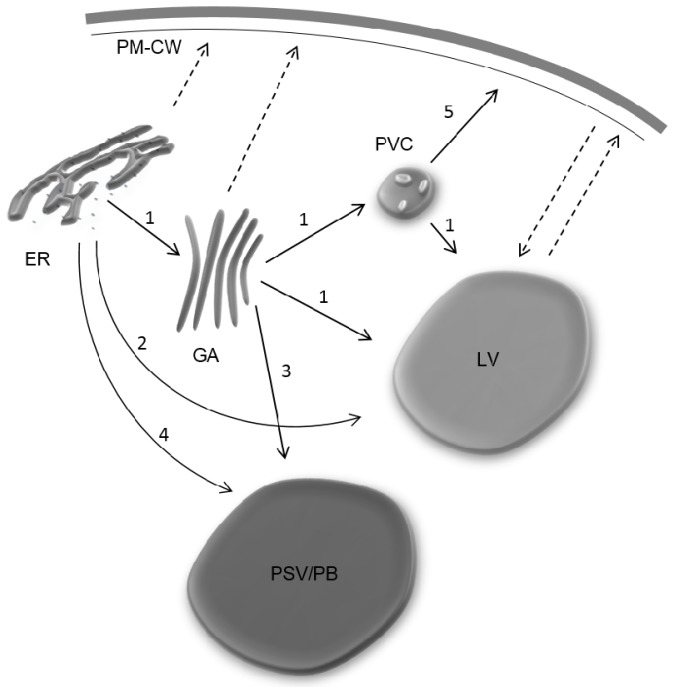
Proposed routes for protein transport to and from the different types of vacuoles. 1, Classical ER-GA route to the lytic vacuole, mainly mediated by *C*-terminal vacuolar sorting determinants (VSDs). This route may, or may not involve the PVC; 2, COPII-independent pathway, sorted by PSI vacuolar sorting determinant without glycosylation site; 3, Putative route from the GA to the vacuole, mediated by PAC (precursor accumulating vesicles) that can be observed in storage organs; 4, Direct route from the ER to the PB/PSV, observed in germinating seeds; 5, Secretion route from the PVC to the PM–CW complex. Dashed arrows represent putative routes that are yet to be confirmed. Abbreviations: ER, endoplasmic reticulum; GA, Golgi apparatus; PM–CW, plasma membrane–cell wall complex; PSV/PB, protein storage vacuole/protein body; PVC, prevacuolar compartment; LV, lytic vacuole.

A model for the proposed trafficking pathways to and from the different compartments, not all necessarily operating in the same cell, is illustrated in the Figure 1.

## 7. Conclusions

Because of the variety in plant models, cell types and experimental approaches used to decipher vacuolar targeting processes, protein trafficking to the vacuole is far from being a closed issue. Novel routes are being disclosed and vacuoles with intermediate characteristics described. Also, the identity of the compartments through which protein are sorted to the vacuole remains elusive. Despite the long established vacuolar signals, some others have been described in the last few years, with different properties that can be specific for some cells or some types of vacuoles. Proteins having two different vacuolar signals have also been described and its significance is not clear although it may point to different sorting depending on the protein roles in a specific context.

The same protein can be either secreted to the apoplast or directed to the vacuole, or accumulate in PSVs or LVs traveling though different routes, depending on the cell type and developmental stage suggesting a tight mechanism of regulation of trafficking. The glycosylation status of the proteins could also interfere with the route taken by the protein to the vacuole.

Such plurality and complexity in sorting mechanisms strengthen the idea of differential vacuolar sorting, suggesting a possible specialization of the trafficking pathways according to the type of cell and its specialization in response to developmental programs, cell identity transitions and stress pathways.

The identification of all required components of vacuolar sorting and its integration in cellular signaling and regulatory networks that regulate membrane traffic in changing developmental and environmental conditions will be the focus of future studies. Systems-level analysis and cell-type specific genomics will likely contribute to the ever-continuing unraveling of the complex issue of vacuolar targeting processes.

## References

[1] Matsuoka K., Nakamura K. (1991). Propeptide of a precursor to a plant vacuolar protein required for vacuolar targeting. Proc. Natl. Acad. Sci. USA.

[2] Martinoia E., Maeshima M., Neuhaus H.E. (2007). Vacuolar transporters and their essential role in plant metabolism. J. Exp. Bot.

[3] Paris N., Stanley C.M., Jones R.L., Rogers J.C. (1996). Plant cells contain two functionally distinct vacuolar compartments. Cell.

[4] Vitale A., Raikhel N.V. (1999). What do proteins need to reach different vacuoles?. Trends Plant Sci.

[5] Paris N., Neuhaus J.M. (2002). Bp-80 as a vacuolar sorting receptor. Plant Mol. Biol.

[6] Olbrich A., Hillmer S., Hinz G., Oliviusson P., Robinson D.G. (2007). Newly formed vacuoles in root meristems of barley and pea seedlings have characteristics of both protein storage and lytic vacuoles. Plant Physiol.

[7] Bolte S., Lanquar V., Soler M.N., Beebo A., Satiat-Jeunemaitre B., Bouhidel K., Thomine S. (2011). Distinct lytic vacuolar compartments are embedded inside the protein storage vacuole of dry and germinating *Arabidopsis thaliana* seeds. Plant Cell Physiol.

[8] Maeshima M., Haranishimura I., Takeuchi Y., Nishimura M. (1994). Accumulation of vacuolar H^+^-pyrophosphatase and H^+^-atpase during reformation of the central vacuole in germinating pumpkin seeds. Plant Physiol.

[9] Inoue K., Motozaki A., Takeuchi Y., Nishimura M., Haranishimura I. (1995). Molecular characterization of proteins in protein-body membrane that disappear most rapidly during transformation of protein bodies into vacuoles. Plant J.

[10] Tohge T., Ramos M.S., Nunes-Nesi A., Mutwil M., Giavalisco P., Steinhauser D., Schellenberg M., Willmitzer L., Persson S., Martinoia E. (2011). Toward the storage metabolome: Profiling the barley vacuole. Plant Physiol.

[11] Neuhaus J.-M., Rogers J. (1998). Sorting of proteins to vacuoles in plant cells. Plant Mol. Biol.

[12] Nebenfuhr A. (2002). Vesicle traffic in the endomembrane system: A tale of cops, rabs and snares. Curr. Opin. Plant Biol.

[13] Richter S., Voss U., Jurgens G. (2009). Post-golgi traffic in plants. Traffic.

[14] Park M., Jurgens G. (2012). Membrane traffic and fusion at post-golgi compartments. Front. Plant Sci.

[15] Foresti O., Gershlick D.C., Bottanelli F., Hummel E., Hawes C., Denecke J. (2010). A recycling-defective vacuolar sorting receptor reveals an intermediate compartment situated between prevacuoles and vacuoles in tobacco. Plant Cell.

[16] Bassham D.C., Raikhel N.V. (2000). Unique features of the plant vacuolar sorting machinery. Curr. Opin. Cell Biol.

[17] Vitale A., Galili G. (2001). The endomembrane system and the problem of protein sorting. Plant Physiol.

[18] Gomez L., Chrispeels M.J. (1993). Tonoplast and soluble vacuolar proteins are targeted by different mechanisms. Plant Cell.

[19] Pereira C.S., da Costa D.S., Pereira S., de Moura-Nogueira F., Albuquerque P.M., Teixeira J., Faro C., Pissarra J. (2008). Cardosins in postembryonic development of cardoon: Towards an elucidation of the biological function of plant aspartic proteinases. Protoplasma.

[20] De Marchis F., Bellucci M., Pompa A. (2013). Traffic of human alpha-mannosidase in plant cells suggests the presence of a new endoplasmic reticulum-to-vacuole pathway without involving the golgi complex. Plant Physiol.

[21] Pereira C., Pereira S., Satiat-Jeunemaitre B., Pissarra J. (2013). Cardosin A contains two vacuolar sorting signals using different vacuolar routes in tobacco epidermal cells. Plant J.

[22] Stigliano E., Faraco M., Neuhaus J.-M., Montefusco A., Dalessandro G., Piro G., di Sansebastiano G.-P. (2013). Two glycosylated vacuolar gfps are new markers for ER-to-vacuole sorting. Plant Physiol. Biochem.

[23] Honig A., Avin-Wittenberg T., Ufaz S., Galili G. (2012). A new type of compartment, defined by plant-specific ATG8-interacting proteins, is induced upon exposure of *Arabidopsis* plants to carbon starvation. Plant Cell.

[24] Robinson D.G., Pimpl P. (2014). Clathrin and post-golgi trafficking: A very complicated issue. Trends Plant Sci.

[25] Viotti C., Krüger F., Krebs M., Neubert C., Fink F., Lupanga U., Scheuring D., Boutté Y., Frescatada-Rosa M., Wolfenstetter S. (2013). The endoplasmic reticulum is the main membrane source for biogenesis of the lytic vacuole in *Arabidopsis*. Plant Cell.

[26] Viotti C. (2014). ER and vacuoles: Never been closer. Front. Plant Sci.

[27] Zouhar J., Rojo E. (2009). Plant vacuoles: Where did they come from and where are they heading?. Curr. Opin. Plant Biol.

[28] Jolliffe N.A., Craddock C.P., Frigerio L. (2005). Pathways for protein transport to seed storage vacuoles. Biochem. Soc. Trans.

[29] Pompa A., de Marchis F., Vitale A., Arcioni S., Bellucci M. (2010). An engineered *C*-terminal disulfide bond can partially replace the phaseolin vacuolar sorting signal. Plant J.

[30] Nakamura K., Matsuoka K. (1993). Protein targeting to the vacuole in plant-cells. Plant Physiol.

[31] Robinson D.G., Oliviusson P., Hinz G. (2005). Protein sorting to the storage vacuoles of plants: A critical appraisal. Traffic.

[32] Nishizawa K., Maruyama N., Utsumi S. (2006). The *C*-terminal region of α′ subunit of soybean β-conglycinin contains two types of vacuolar sorting determinants. Plant Mol. Biol.

[33] Simões I., Faro C. (2004). Structure and function of plant aspartic proteinases. Eur. J. Biochem.

[34] Holkeri H., Vitale A. (2001). Vacuolar sorting determinants within a plant storage protein trimer act cumulatively. Traffic.

[35] Rayon C., Lerouge P., Faye L. (1998). The protein *N*-glycosylation in plants. J. Exp. Bot.

[36] Paris N., Saint-Jean B., Faraco M., Krzeszowiec W., Dalessandro G., Neuhaus J.M., di Sansebastiano G.P. (2010). Expression of a glycosylated gfp as a bivalent reporter in exocytosis. Plant Cell Rep.

[37] Wilkins T.A., Bednarek S.Y., Raikhel N.V. (1990). Role of propeptide glycan in posttranslational processing and transport of barley lectin to vacuoles in transgenic tobacco. Plant Cell.

[38] Hara-Nishimura I., Shimada T., Hatano K., Takeuchi Y., Nishimura M. (1998). Transport of storage proteins to protein storage vacuoles is mediated by large precursor-accumulating vesicles. Plant Cell.

[39] Oliveira A., Pereira C., da Costa D.S., Teixeira J., Fidalgo F., Pereira S., Pissarra J. (2010). Characterization of aspartic proteinases in *C. cardunculus* L. callus tissue for its prospective transformation. Plant Sci.

[40] Duarte P., Pissarra J., Moore I. (2008). Processing and trafficking of a single isoform of the aspartic proteinase cardosin a on the vacuolar pathway. Planta.

[41] RamalhoSantos M., Pissarra J., Verissimo P., Pereira S., Salema R., Pires E., Faro C.J. (1997). Cardosin A, an abundant aspartic proteinase, accumulates in protein storage vacuoles in the stigmatic papillae of *Cynara cardunculus* L. Planta.

[42] Vieira M., Pissarra J., Verissimo P., Castanheira P., Costa Y., Pires E., Faro C. (2001). Molecular cloning and characterization of CDNA encoding cardosin B, an aspartic proteinase accumulating extracellularly in the transmitting tissue of *Cynara cardunculus* L. Plant Mol. Biol.

[43] Xiang L., Etxeberria E., van den Ende W. (2013). Vacuolar protein sorting mechanisms in plants. FEBS J.

[44] De Caroli M., Lenucci M.S., di Sansebastiano G.P., Dalessandro G., de Lorenzo G., Piro G. (2011). Protein trafficking to the cell wall occurs through mechanisms distinguishable from default sorting in tobacco. Plant J.

[45] Duarte P., Figueiredo R., Pereira S., Pissarra J. (2006). Structural characterization of the stigma-style complex of *Cynara cardunculus* (asteraceae) and immunolocalization of cardosins A and B during floral development. Can. J. Bot.

[46] Rose J.K., Lee S.J. (2010). Straying off the highway: Trafficking of secreted plant proteins and complexity in the plant cell wall proteome. Plant Physiol.

[47] Drakakaki G., Dandekar A. (2013). Protein secretion: How many secretory routes does a plant cell have?. Plant Sci.

[48] Ding Y., Wang J., Stierhof Y.D., Robinson D.G., Jiang L. (2012). Unconventional protein secretion. Trends Plant Sci.

